# The Relation between Self-Reported Empathy and Motor Identification with Imagined Agents

**DOI:** 10.1371/journal.pone.0014595

**Published:** 2011-01-26

**Authors:** Daniele Marzoli, Rocco Palumbo, Alberto Di Domenico, Barbara Penolazzi, Patrizia Garganese, Luca Tommasi

**Affiliations:** 1 Department of Biomedical Sciences and Advanced Therapy, University of Ferrara, Ferrara, Italy; 2 Department of Neuroscience and Imaging, University G. d'Annunzio, Chieti, Italy; 3 Department of Psychology, University Alma Mater Studiorum, Bologna, Italy; The University of Western Ontario, Canada

## Abstract

**Background:**

In a previous study, we found that when required to imagine another person performing an action, participants reported a higher correspondence between their own handedness and the hand used by the imagined person when the agent was seen from the back compared to when the agent was seen from the front. This result was explained as evidence of a greater involvement of motor areas in the back-view perspective, possibly indicating a greater proneness to put oneself in the agent's shoes in such a condition. In turn, the proneness to put oneself in another's shoes could also be considered as a cue of greater identification with the other, that is a form of empathy. If this is the case, the proportion of lateral matches vs mismatches should be different for subjects with high and low self-reported empathy. In the present study, we aimed at testing this hypothesis.

**Methodology/Principal Findings:**

Participants were required to imagine a person performing a single manual action in a back view and to indicate the hand used by the imagined person during movement execution. Consistent with our hypothesis, the proportion of matching between the handedness of participants and the handedness of agents imagined was higher for participants scoring high in a self-report measure of empathy. Importantly, this relationship was specific for females.

**Conclusions/Significance:**

At least for females, our data seem to corroborate the idea of a link between self-reported empathy and motor identification with imagined agents. This sex-specific result is consistent with neuroimaging studies indicating a stronger involvement of action representations during emotional and empathic processing in females than in males. In sum, our findings underline the possibility of employing behavioral research as a test-bed for theories deriving from functional studies suggesting a link between empathic processing and the activation of motor-related areas.

## Introduction

In general, the term empathy refers to the process of understanding others' mental and emotional states and reacting to them appropriately, and involves both cognitive and emotional dimensions [1], [2]. According to Gallese [3], the establishment of a self-other equivalence is crucial for the cognitive development of complex forms of interpersonal relationships, including empathy. Gallese proposed that some degree of identity is crucial in social species, because it allows individuals to better predict the consequences of others' behavior: specifically, the attribution of identity status to other subjects reduces the amount of information that the brain has to process. Indeed, the same neural structures involved in processing one's own actions, sensations and emotions, are thought to be active when those actions, sensations and emotions are observed in others [4]. In line with this proposal, many studies suggest that empathy might rely on an automatic activation of the motor representation of the observed action [5]–[7]. Additional evidence for the involvement of the motor system in empathic processes is offered by studies indicating that the observation of painful stimulations delivered to others is related to the simulation of sensorimotor aspects of others' experience, and that such a modulation correlates positively with both the intensity of the pain attributed to the observed model and the empathic tendencies of the observer [8]–[12] (see [13] for consistent findings in a study engaging participants with Asperger syndrome, a disorder characterized by reduced or lacking empathy).

The notion of an automatic activation of motor representations in empathic processes is further corroborated by the significant association observed between self-reports of empathy and neural activity in regions of the mirror neuron system (MNS) during different tasks, and particularly during tasks involving emotion and pain processing [14]–[21]. It has also been proposed that motor simulation may be involved more in emotional than in cognitive empathy [22], although a specific association between activity in the MNS and self-reports of emotional rather than cognitive empathy has not been demonstrated yet [16].

Finally, there is some evidence that impairments in empathizing and/or mentalizing skills are related to deficits in visuospatial perspective taking [23], [24], and recent research suggests a relationship between self-reported empathy and visuospatial processing as measured by imagined self-other transformations, biases in spatial attention and mental rotation of letters [25]–[27].

It is worth noting that the positive association between empathic abilities and the recruitment of motor representations is supported by much functional neuroimaging research, but few behavioral studies have addressed this issue (e.g., [28]; see [29], [30] for consistent EMG results). The present investigation aims at shedding more light on this topic. In a previous study [31], we found that when required to imagine another person performing an action, participants reported a higher correspondence between their own handedness and the hand used by the imagined agent when the agent was seen from the back compared to when the agent was seen from the front. This front-back difference seems to be consistent with research on mental spatial transformation of human bodies and body parts, which indicates 1) faster left–right judgements about human bodies for figures presented with a back view than with a front view, and 2) a crucial interaction between motor simulation and hand dominance during the perception of bodies and body parts [32]–[37]. In line with studies indicating an overlap between neural structures involved in action production and in both self- and other-related action imagination (e.g., [38]–[41]; for a review, see [42]), we interpreted our result [31] as evidence of a greater involvement of motor areas in the back-view perspective (which we assume to be more readily assimilable to an egocentric view; e.g., [43]), maybe indicating a greater proneness to put oneself in the other's shoes in such a condition. In this regard, we point out that differential effects of viewing perspectives have been observed for the activation of the MNS during the observation of hands playing a competitive game [44], the somatosensory cortices during the observation of touch [45], the extrastriate body area during the observation of bodies and body parts [46], [47] and the temporoparietal junction during the mental imagery of one's own body [48], [49], as well as for the illusion of body ownership in virtual reality [50].

In our opinion, the proneness to put oneself in another's shoes could also be considered as a cue of greater identification with the other, that is a form of empathy [1]. If this is the case, the proportion of lateral matches vs mismatches during the imagination of others' actions should be different for subjects with high and low self-reported empathy. Specifically, assuming that a greater involvement of motor areas would result in higher correspondence between one's own handedness and the hand used by the imagined agent, our prediction is strongly supported by the aforementioned studies indicating a significant link between empathy and the activation of motor representations. In order to test our hypothesis, we devised a study in which participants were asked to imagine a person – seen from behind – performing a manual action and then to indicate the hand used by the imagined agent by showing their own right or left hand. In this study we examined exclusively the back-view perspective because the involvement of motor representations is assumed to be stronger compared to the front-view condition [31], [43]. Participants were also required to complete the Interpersonal Reactivity Index (IRI; [51]) and the Balanced Emotional Empathy Scale (BEES; [52]), two of the most frequently used questionnaires to assess empathy.

## Methods

### Imagination task

One of four experimenters (two females and two males) approached 376 subjects (188 females and 188 males) aged approximately 20–30 years in various locations on the University campus, malls and other places where they could sit comfortably. Since neither invasive nor risky procedures were involved and since the data were analyzed anonymously, participants were required to give only oral consent. The study was carried out in accordance with the principles of the Declaration of Helsinki and following the approval of the local ethical committee (Comitato Etico d'Ateneo, Università “G. d'Annunzio” – Chieti).

The experimenter, standing in front of the participant, made sure that the subject placed her/his hands palm-down on the knees or on a table, and that she/he did not cross her/his legs, arms or even fingers. Then the experimenter invited the participant to close her/his eyes and to imagine a female or male person seen from behind, without giving any further clue on the identity of the person to be imagined. Once the participant stated to ‘see’ the person, the experimenter asked her/him to imagine that the person was performing an action. Thereafter, when the participant stated to ‘see’ the person's movement clearly, the experimenter asked her/him to open her/his eyes and to indicate the hand used by the person by showing her/his own right or left hand. Only one trial was administered to each participant. The trial took around 3–4 minutes to be accomplished. In order to assess the participant's hand preference, she/he was administered the Italian version of the Edinburgh Handedness Inventory [53], which measures laterality as a continuous variable ranging from -1 to +1 (from complete left-handedness to complete right-handedness).

Three groups of participants were asked to imagine a person performing one of these three actions: using scissors (120 subjects), using a toothbrush (112 subjects) or using a spoon (144 subjects). Each group was composed of an identical number of:

- females imagining a female person;
- females imagining a male person;
- males imagining a female person;
- males imagining a male person.

Only subjects who were able to ‘see’ the person and the person's movement and to clearly indicate a definite (right or left) hand for the imagined action were included in the study. Of all subjects who succeeded in performing the task, none spontaneously reported having imagined her/himself as the person performing the movement, and only few subjects reported having imagined a familiar person.

### Self-reported empathy

After completing the handedness questionnaire, participants were administered the Italian versions of the Interpersonal Reactivity Index (IRI; [54]) and the Balanced Emotional Empathy Scale (BEES; [55]).

The IRI [51] is a 28-item questionnaire consisting of four discrete, seven-item subscales assessing different dimensions of empathy: Perspective Taking (PT), Fantasy (FS), Empathic Concern (EC), and Personal Distress (PD).

The BEES [52] is a 30-item questionnaire that assesses the tendency to share the emotional experiences of others, and represents a measure of emotional empathy.

More detailed descriptions of both questionnaires are available as Supporting Information (Text S1).

### Data analysis

According to the laterality score obtained in the Italian version of the Edinburgh Handedness Inventory [53], the 376 participants were divided into two categories: right-handers (345 subjects with a positive laterality score [range: 0.04/1.00; M = 0.67±0.013 SEM]) or left-handers (30 subjects with a negative laterality score [range: −1.00/−0.03; M = −0.55±0.052 SEM]). One female participant with a laterality score equal to 0 was excluded from data analyses.

As in our previous study [31], the proportion of matches vs mismatches between participants' dominant hand and the hand used by the imagined agent did not differ across actions (χ^2^ = 1.623, d.f. = 2, p = 0.444; Table 1), so we did not consider the variable Action in the following analyses.

**Table 1:** Proportion of matches vs mismatches between participants' dominant hand and the hand used by the imagined agent for the different actions.

Action	Matches	Mismatches
Using scissors	102	17
Using a toothbrush	102	10
Using a spoon	126	18

#### Imagination task

Chi-square tests were used 1) to compare the proportion of matches vs mismatches between the handedness of participants and the handedness of the imagined agent in right- and left-handed participants and 2) to examine the possible effect of both the participants' sex and the correspondence between the participants' sex and the imagined agent's sex on the proportion of lateral matches vs mismatches.

#### IRI susbscales

In addition to the female participant with a laterality score equal to 0, in the analyses of the IRI scores we excluded 24 female participants and 26 male participants who either did not respond to one or more items of any IRI subscale or scored more than 2 standard deviations above or below the mean in any IRI subscale according to their ‘Sex x Lateral Correspondence’ group (i.e., females and males who imagined the action being performed with their dominant or non-dominant hand).

A multivariate analysis of variance was performed on all IRI subscales: PT, FS, EC and PD. The independent variables were Participant's Sex (female, male) and Lateral Correspondence between the participant's dominant hand and the hand used by the imagined agent (same hand, different hand). Because of the low number of left-handed participants (n = 20) available for this analysis, it was not possible to include handedness (i.e., left or right manual dominance) as an independent variable. Consequently, Laterality Score (as measured by the Italian version of the Edinburgh Handedness Inventory) was included as a covariate.

#### BEES

In addition to the female participant with a laterality score equal to 0, in the analyses of the BEES scores we excluded 9 female participants and 14 male participants who either did not respond to one or more items or scored more than 2 standard deviations above or below the mean according to their ‘Sex x Lateral Correspondence’ group (i.e., females and males who imagined the action being performed with their dominant or non-dominant hand).

A univariate analysis of variance was performed on the BEES. The independent variables were Participant's Sex (female, male) and Lateral Correspondence between the participant's dominant hand and the hand used by the imagined agent (same hand, different hand). Because of the low number of left-handed participants (n = 27) available for this analysis, it was not possible to include handedness (i.e., left or right manual dominance) as an independent variable. Consequently, Laterality Score (as measured by the Italian version of the Edinburgh Handedness Inventory) was included as a covariate.

## Results

### Imagination task

Compared to a chance distribution (50%), right-handers imagined a higher proportion of right- than left-handed actions (313 vs 32 [90.7%]; χ^2^ = 228.872, d.f. = 1, p<0.001), while left-handers imagined a larger proportion of left- than right-handed actions, although this difference was not significant (17 vs 13 [56.7%]; χ^2^ = 0.533, d.f. = 1, p = 0.465). Moreover, right-handers showed a larger proportion of matches vs mismatches between their dominant hand and the hand used by the imagined agent compared to left-handers (χ^2^ = 27.177 [Continuity Correction Applied, from now on: CCA], d.f. = 1, p<0.001).

The proportion of matches vs mismatches did not differ according to either the participants' sex (females: 162 vs 25 [86.6%]; males: 168 vs 20 [89.4%]; χ^2^ = 0.429 [CCA], d.f. = 1, p = 0.513) or the correspondence between the participants' sex and the imagined agent's sex (same sex: 167 vs 21 [88.8%]; opposite sex: 163 vs 24 [87.2%]; χ^2^ = 0.113 [CCA], d.f. = 1, p = 0.736).

When participants were divided according to their lateral preference for the specific action imagined (using scissors, using a toothbrush, or using a spoon) as declared in the handedness questionnaire rather than total laterality score obtained by considering all items, results were almost identical. However, this choice would have entailed the loss of 30 participants who indicated no lateral preference for the particular action imagined, so we decided to report only the results of analyses employing total laterality score as the criterion for defining handedness.

### IRI susbscales

**Figure 1:**
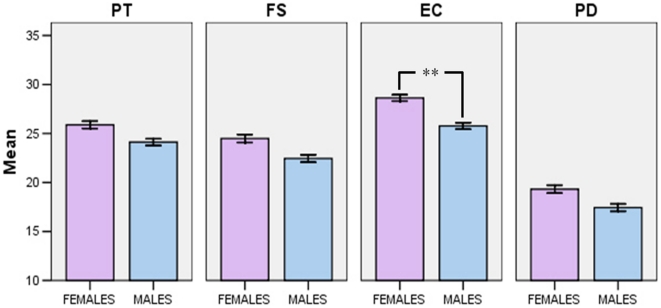
Female and male participants' scores on the IRI subscales (PT: Perspective Taking; FS: Fantasy; EC: Empathic Concern; PD: Personal Distress).

The only significant effect was that of Sex (F_4,317_ = 3.281; p<0.05). Post hoc univariate analyses showed that females (n = 163) scored significantly higher than males (n = 162) in the EC subscale (M_f_ = 28.63 vs M_m_ = 25.77; F_1,320_ = 10.472; p<0.005), and a statistical trend in the same direction was observed in the PD subscale (M_f_ = 19.32 vs M_m_ = 17.44; F_1,320_ = 3.108; p = 0.079), while there were no differences in the PT subscale (M_f_ = 25.89 vs M_m_ = 24.12; F_1,320_ = 1.165; p = 0.281) and in the FS subscale (M_f_ = 24.49 vs M_m_ = 22.45; F_1,320_ = 2.368; p = 0.125; Figure 1).

### BEES

Statistical analyses showed significant main effects of both Sex (F_1,347_  =  13.798; p<0.001) and Lateral Correspondence (F_1,347_ = 4.023; p<0.05): females scored higher (n = 178; M = 37.56) than males (n = 174; M = 18.93), and participants who imagined the action being performed with their dominant hand scored higher (n = 309; M = 29.17) than those who imagined the action being performed with their non-dominant hand (n = 43; M = 22.44).

**Figure 2:**
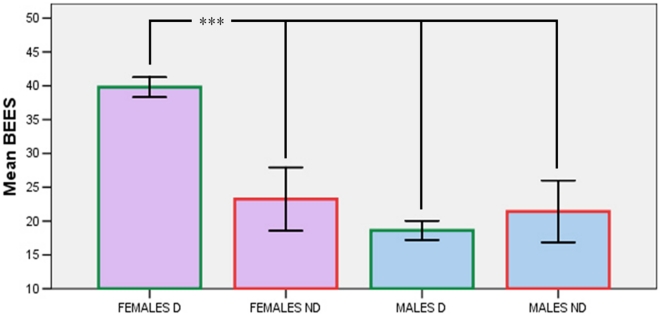
BEES scores as a function of Sex and Lateral Correspondence (D: dominant hand; ND: non-dominant hand).

A significant interaction between Sex and Lateral Correspondence (F_1,347_ = 10.467; p<0.005) showed that the previous results were due to females who imagined the action being performed with their dominant hand scoring significantly higher (n = 154; M = 39.79) than all the other groups (females, non-dominant: n = 24, M = 23.25; males, dominant: n = 155, M = 18.62; males, non-dominant: n = 19, M = 21.42; all ps<0.001; p values adjusted with the Tukey-Kramer method), while there were no differences between these latter groups (Figure 2).

## Discussion

### Imagination task

With regard to participants' responses in the imagination task, the present results confirm those of our previous study [31]: 1) both right-handers and left-handers preferentially imagined actions performed with their dominant rather than non-dominant hand (although this difference was significant only for right-handers, possibly because the number of left-handed participants was not sufficient to detect a rather moderate difference); 2) compared to left-handers, right-handers reported a larger proportion of matches between their dominant hand and the hand used by the imagined agent. As already proposed [31], the lower proportion of matches vs mismatches in left-handers may be attributed to both their greater visual familiarity with actions performed with their non-dominant hand (inducing an incongruence between the visual experience of others' actions and one's own experience of self-made actions) and their lower average absolute degree of lateralization, in line with previous research showing handedness-related differences with regard to motor representations in both behavioral and functional measures[56]–[58].

### Self-reported empathy

Compared to males, females exhibited higher empathy scores, and the difference was particularly significant for the emotional component of empathy, as apparent in participants' responses to the IRI EC subscale and the BEES. This is consistent with past research employing self-report data [59]–[61].

Our results provide good (albeit not complete) support for the hypothesis that participants imagining actions performed with their dominant hand would exhibit higher empathy compared to those imagining actions performed with their non-dominant hand. In fact, the effect of lateral correspondence was due exclusively to women, because female participants who imagined actions performed with their dominant hand scored higher on the BEES than all other groups, which did not differ between each other.

If one assumes that the lateral congruence between one's own and an imagined agent's handedness can be considered as indicative of a greater involvement of motor areas compared to the lateral incongruence, our results are consistent with previous research suggesting a link between empathic tendencies and the activation of motor-related areas [14]–[19], [28]–[30]. In particular, it is important to note that much research indicates a stronger activation of motor representations during emotional and empathic processes in women than in men. For example, females exhibit more pronounced and more congruent facial electromyographic reactions to observed facial expressions than males [62]–[64]. Compared to men, women show enhanced MNS activation during emotional perspective taking, which may be related to gender differences in the tendency to exhibit facial mimicry [65]. Moreover, during the simple observation of leg and hand movements as well as during the observation of body parts in both painful and non-painful situations, activity in the MNS (as indicated by mu rhythm suppression) is stronger in females than in males [66]–[69]. Interestingly, there is some evidence that such activity is positively correlated with self-reports of empathy in females but not in males (i.e., painful condition in [69]; for congruent findings, see [70]). Overall, these studies strongly suggest that females activate motor-related areas more than males during emotional and empathic processing, consistent with neuroimaging research indicating that females and males rely on different neural strategies for these tasks (e.g., [71]–[75]).

Furthermore, females and males report using respectively egocentric strategies and object-based strategies when performing visuospatial perspective-taking tasks, and these preferences appear to be associated with the recruitment of specific brain regions [26], [76]. Congruent differences are also found for egocentric mental rotation of hands, females relying more on imitation or perceptual comparisons (as suggested by stronger activity in the left ventral premotor cortex), and males relying more on early visual or semantic processing (as suggested by stronger activity in the lingual gyrus) [77]. Moreover, females seem to engage more emotion and self-related processes and males more cognitive processes during tasks involving both affective and cognitive empathy, even in the absence of significant gender differences in behavioral performance [78]. Finally, an intriguing instance of more strongly embodied representations in females than in males can be observed in giving route directions, a task in which women are more concrete and personal, using landmarks and left–right terms, whereas men are more abstract and Euclidean, using cardinal directions and metric distances [79] (see also [80], for an interesting correlation between anxiety during environmental navigation and way-finding strategies). In sum, it is plausible that the gender-specific result found in our study may be a consequence of the stronger involvement, in more empathic females, of motor- and self-related processes, which could result in more body-specific (see [81]) and ‘concrete’ imagery.

Noteworthy, a significant link between the hand used by the imagined agent and self-reports of empathy was found when examining the BEES [52] scores, but not when examining any IRI [51] subscale scores. This result makes sense if one considers that different dimensions of empathy are measured by these instruments. In fact, as regards the IRI [51], while the PT and FS subscales assess cognitive empathy, the EC subscale assesses the tendency to sympathize with and be concerned about others, and the PD subscale assesses the tendency to feel negative emotions in response to others' distress. On the other hand, the BEES [52] assesses the tendency to vicariously experience both positive and negative emotions of others, and thus could provide a more specific measure of affective sharing. Therefore, compared to any IRI subscale, the BEES might prove to be a more sensitive marker of the tendency to engage motor representations, because affective empathy seems to activate motor-related areas more intensely than cognitive empathy [22].

Since, in line with some studies reporting a left hemisphere dominance for empathic abilities in right-handers [26], [82], we observed a positive correlation between female participants' laterality scores and BEES scores (see Supporting Information, Text S2 and Figure S1) and, in line with our previous study [31], absolute laterality score was positively associated with the likelihood to imagine an action performed with one's own dominant rather than non-dominant hand (see Supporting Information, Text S3), the positive association between lateral correspondence and the BEES scores observed in our female participants might be attributed to the covariance of both these variables with laterality scores, the great majority of our participants being right-handed. However, this possibility can be ruled out because a significant interaction between Sex and Lateral Correspondence was also found when the analysis (employing Laterality Score as a covariate) was carried out for right-handers only (see Supporting Information, Text S3 and Figure S2).

A possible concern regarding the procedure used to investigate our hypotheses could consist in the fact that we asked our subjects to ‘see’ an imagined person performing an action, which might seemingly engage visual rather than motor imagery. Since visual imagery is usually associated with brain areas – mainly not motor – different from those associated with motor imagery (e.g., [83], [84]), one could wonder why a visual imagery task should have tapped motor representations (and thus the allegedly motor neural basis of empathic abilities). However, we point out that, as well as during others' action perception (see [42] for a review), the activation of motor representations is found during the imagination of not only one's own (e.g., [39], [41]) but also of others' movements (e.g., [40]). In particular, as regards the link between empathic abilities and the recruitment of motor representations, a positive correlation between self-reports of empathy and neural activity in regions of the MNS has been reported for different tasks, including tasks which might involve mainly visual representations (i.e., the observation of pleasant and unpleasant emotions [17]) or even the mere imagination of others' behavior (i.e., the anticipation of others' emotional responses [15]). So, it is plausible that empathic tendencies could affect performance in an imagination task which is deemed to involve one's own motor representations [40] (see also [31] for consistent behavioral results).

In conclusion, the present study corroborates our hypothesis that self-reported empathy is positively linked to the activation of motor representations while imagining another person performing an action. However, this relationship seems to be present only in female subjects, consistent with previous research indicating a stronger involvement of action representations during emotional and empathic processing in females than in males [62]–[65]. In other words, compared to male participants, female participants were likely to use more self- and motor-related representations during the imagination task, which is consistent with neuroimaging findings on gender-specific neural activity during both visuospatial and emotional perspective taking [65], [76], [78].

Finally, our results underline the possibility of employing behavioral research as a test-bed for theories deriving from functional studies. Conversely, a possible extension of this study could consist in employing neuroimaging techniques such as fMRI and TMS in order to test the proposed correlation between self-reported empathy and body-specific motor representations [81] during others' action imagination.

## Supporting Information

Text S1. Empathy questionnaires description.
(0.04 MB DOC)

Text S2. Correlation between empathic tendencies and hemispheric lateralization.
(0.04 MB DOC)

Text S3. BEES analysis for right-handed participants.
(0.04 MB DOC)

Figure S1. Scatterplots of Laterality Score and BEES for female and male right-handed participants.
(1.88 MB TIF)

Figure S2. BEES scores as a function of Sex and Lateral Correspondence (D: dominant hand; ND: non-dominant hand) for right-handed participants.
(0.77 MB TIF)
