# Supplementary material for: The Relation between Self-Reported Empathy and Motor Identification with Imagined Agents
Source: PLoS One. 2011 Jan 26;6(1):e14595. doi: 10.1371/journal.pone.0014595 (PMC3027625; doi:10.1371/journal.pone.0014595)
Supplement: Text S1. — Empathy questionnaires description. (0.04 MB DOC) [file pone.0014595.s001.doc]

**Supporting Information**

Text S1

*Empathy questionnaires description*

The IRI [51] is a 28-item questionnaire consisting of four discrete, seven-item subscales assessing different dimensions of empathy: Perspective Taking (PT), Fantasy (FS), Empathic Concern (EC), and Personal Distress (PD). This questionnaire was developed using a multidimensional approach and is used to evaluate both the cognitive and affective components of empathy. The FS scale assesses the tendency to imaginatively transpose oneself into fictional characters such as those in books and movies. The PT scale assesses the tendency to adopt the point of view of others (the items included in this scale refer not to fictitious characters or situations, but to "real life" situations). The EC scale assesses the tendency to sympathize with and be concerned about others in need. The PD scale assesses the tendency to feel negative emotions in response to others’ distress. The FS and PT scales measure the cognitive component of empathy, while the EC and PD scales measure the emotional component of empathy. Our participants were administered the Italian version of the IRI [54]: each item is presented as a statement, and participants express their agreement or disagreement on a 5-point Likert scale ranging from 1 (“does not describe me well”) to 5 (“describes me well”).

The BEES [52] is a 30-item questionnaire that assesses the tendency to share the emotional experiences of others, and represents a measure of emotional empathy. It is designed to balance the tendency of most people to generally agree with all the statements included in a questionnaire, so 15 items are phrased as positive statements and 15 as negative statements. Our participants were administered the Italian version of the BEES [55]: each item is presented as a statement, and participants express their agreement or disagreement on a 7-point Likert scale ranging from -3 (‘‘completely agree’’) to +3 (‘‘completely disagree’’). The total score is calculated as the difference between the sum of scores assigned to positively worded items and the sum of scores assigned to negatively worded items.
