# Supplementary material for: The Relation between Self-Reported Empathy and Motor Identification with Imagined Agents
Source: PLoS One. 2011 Jan 26;6(1):e14595. doi: 10.1371/journal.pone.0014595 (PMC3027625; doi:10.1371/journal.pone.0014595)
Supplement: Text S2. — Correlation between empathic tendencies and hemispheric lateralization. (0.04 MB DOC) [file pone.0014595.s002.doc]

**Supporting Information**

Text S2

*Correlation between empathic tendencies and hemispheric lateralization*

Some studies reported a left hemisphere dominance for empathic abilities in right-handers [26,82]. In particular, Thakkar et al.’s [26] observed that in right-handed individuals the score on the IRI EC subscale is positively associated with a rightward attentional bias (as assessed by a line bisection task), and proposed that this might be linked to the left hemisphere involvement in prosocial behaviors [S1]. Although the tasks used in the present study only allow little inference about the relation between hemispheric lateralization and empathy, we wondered whether an association between empathy and laterality score was present in our right-handed participants, so a series of correlational analyses was performed between Laterality Score and both the four IRI subscales and the BEES.

A significant positive correlation was observed only between Laterality Score and BEES (n = 337; r = 0.149; p < 0.05, adjusted for 5 comparisons with the Bonferroni method), which held true for females (n = 169; r = 0.275; p < 0.005, adjusted for 10 comparisons with the Bonferroni method) but not for males (n = 168; r = -0.002; p = 1, adjusted for 10 comparisons with the Bonferroni method) when females and males were examined separately (Figure S1).

Thus, right-handed female participants’ BEES scores correlated positively with their laterality scores, which is consistent with [26]. On the other hand, our results are seemingly at odds with other behavioral studies showing a positive correlation between self-reported empathy and right-hemispheric activity [S2,S3]. However, this apparent incongruence could be due to the use of different tasks in each study.

In our sample, the association between self-reported empathy and laterality score turned out to be gender-specific, because it was observed in females but not in males when each gender was analyzed separately (see [S2] for an analogous result). This finding further supports the notion that empathic processes engage different brain regions in females and males.

*Supporting references*

[S1] Buck R (1999) The biological affects: A typology. Psychol Rev 106: 301–336.

[S2] Rueckert L, Naybar N (2008) Gender differences in empathy: The role of the right hemisphere. Brain Cognition 67: 162–167.

[S3] Spinella M (2002) A relationship between smell identification and empathy. Int J Neurosci 112: 605–612.
