# Supplementary material for: The Relation between Self-Reported Empathy and Motor Identification with Imagined Agents
Source: PLoS One. 2011 Jan 26;6(1):e14595. doi: 10.1371/journal.pone.0014595 (PMC3027625; doi:10.1371/journal.pone.0014595)
Supplement: Text S3. — BEES analysis for right-handed participants. (0.04 MB DOC) [file pone.0014595.s003.doc]

**Supporting Information**

Text S3

*BEES analysis for right-handed participants*

Since participants’ laterality scores correlated positively with their BEES scores (see Text S2 and Figure S1) and, in line with our previous study [31], absolute laterality score was higher in participants who imagined actions performed with their own dominant (M = 0.67) rather than non-dominant hand (M = 0.59; t(373) = 2.101, p(one-tailed) < 0.05), the positive association between lateral correspondence and the BEES scores observed in our female participants might be attributed to the covariance of both variables with laterality scores, the great majority of our participants being right-handed. In order to dispel this doubt, a univariate analysis of variance was performed on the BEES with right-handed participants only. Also in this case, the independent variables were Participant’s Sex (female, male) and Lateral Correspondence between the participant’s dominant hand and the hand used by the imagined person (same hand, different hand), and again Laterality Score (as measured by the Italian version of the Edinburgh Handedness Inventory) was included as a covariate. As for the analysis including left-handed participants, we excluded participants (8 females and 12 males) who either did not respond to one or more items or scored more than 2 standard deviations above or below the mean according to their ‘Sex x Lateral Correspondence’ group (i.e., females and males who imagined the action being performed with their dominant or non-dominant hand).

The only significant effect was the interaction between Sex and Lateral Correspondence (F_1,320_ = 15.183; p < 0.001): females who imagined the action being performed with their dominant hand scored higher (n = 147; M = 39.59) than all the other groups (females, non-dominant: n = 18, M = 19.33, p < 0.001; males, dominant: n = 148, M = 18.63, p < 0.001; males, non-dominant: n = 12, M = 25.50, p = 0.054; p values adjusted with the Tukey-Kramer method; Figure S2). This result rules out the possibility that the positive association between lateral correspondence and the BEES scores observed in our female participants may be due to the covariance of these variables with laterality scores in right-handed participants.
