# Supplementary figures and images for: The Relation between Self-Reported Empathy and Motor Identification with Imagined Agents
Source: PLoS One. 2011 Jan 26;6(1):e14595. doi: 10.1371/journal.pone.0014595 (PMC3027625; doi:10.1371/journal.pone.0014595)

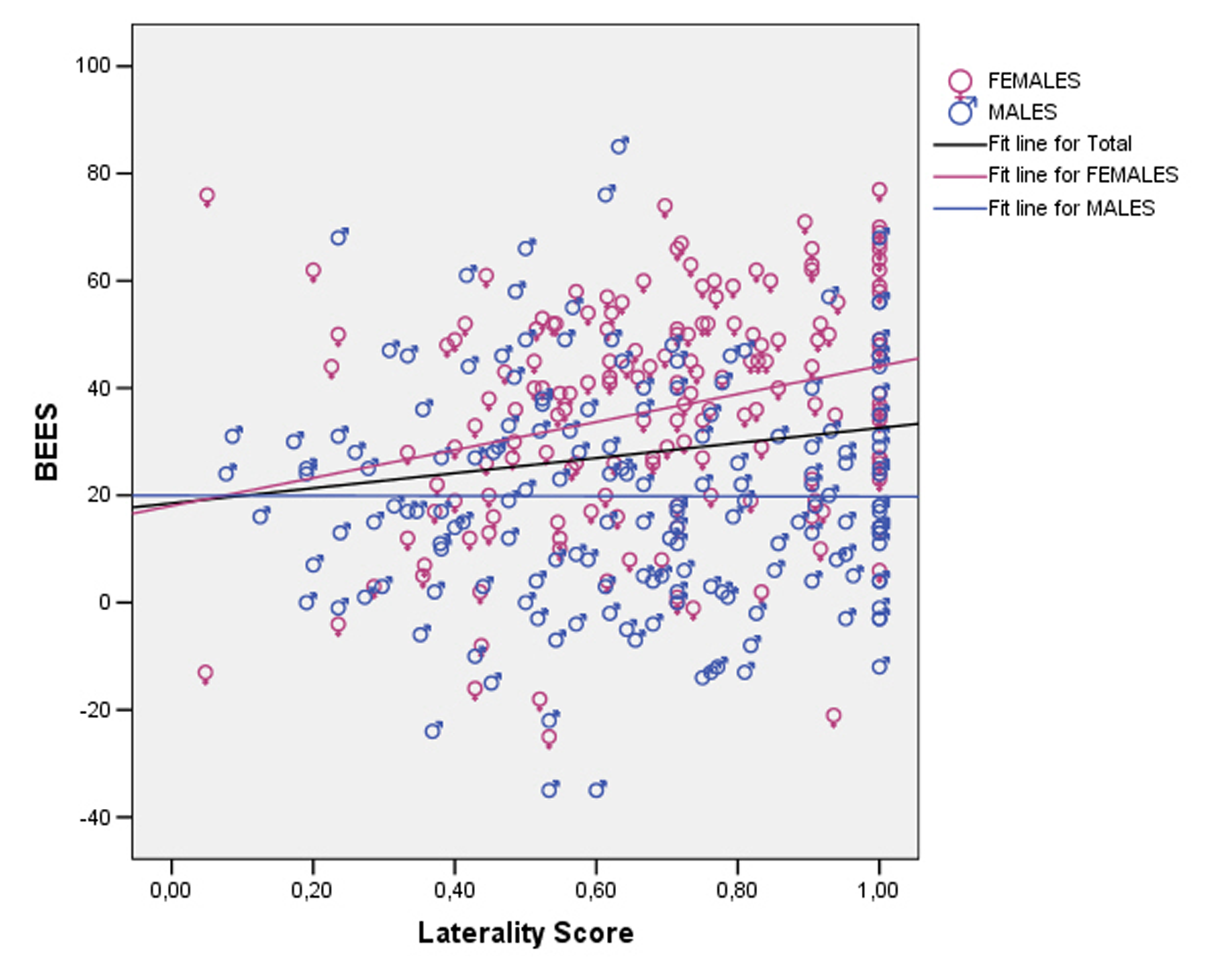

Supplement: Figure S1. — Scatterplots of Laterality Score and BEES for female and male right-handed participants. (1.88 MB TIF) [file pone.0014595.s004.tif]

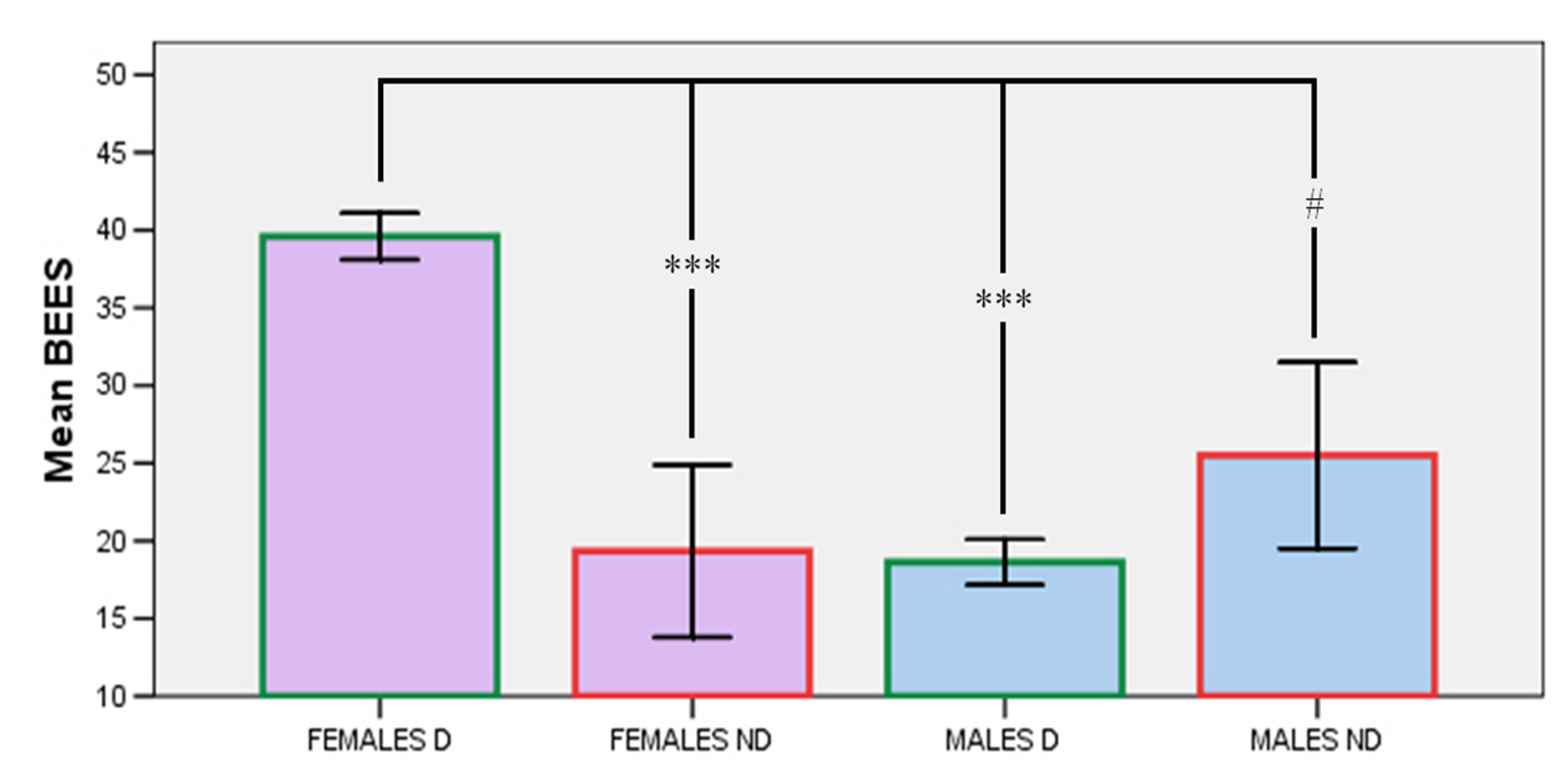

Supplement: Figure S2. — BEES scores as a function of Sex and Lateral Correspondence (D: dominant hand; ND: non-dominant hand) for right-handed participants. (0.77 MB TIF) [file pone.0014595.s005.tif]
